# Treatment-induced cell cycle kinetics dictate tumor response to chemotherapy

**DOI:** 10.18632/oncotarget.3140

**Published:** 2015-02-07

**Authors:** Robin M. Hallett, Cheng Huang, Ali Motazedian, Stefanie Auf der Mauer, Gregory R. Pond, John A. Hassell, Robert E. Nordon, Jonathan S. Draper

**Affiliations:** ^1^ McMaster Stem Cell and Cancer Research Institute, Michael G. DeGroote School of Medicine, McMaster University, Hamilton, ON L8N 3Z5, Canada; ^2^ Department of Pathology and Molecular Medicine, McMaster University, Hamilton, ON L8N 3Z5, Canada; ^3^ Department of Biochemistry and Biomedical Sciences, McMaster University, Hamilton, ON L8N 3Z5, Canada; ^4^ Graduate School of Biomedical Engineering, University of New South Wales, Sydney 2052, Australia; ^5^ Department of Oncology, McMaster University, Hamilton, ON L8N 3Z5, Canada; ^6^ Murdoch Children's Research Institute, The Royal Children's Hospital, Parkville, Victoria 3052, Australia

**Keywords:** Breast cancer, chemotherapy, cell cycle, p53

## Abstract

Chemotherapy fails to provide durable cure for the majority of cancer patients. To identify mechanisms associated with chemotherapy resistance, we identified genes differentially expressed before and after chemotherapeutic treatment of breast cancer patients. Treatment response resulted in either increased or decreased cell cycle gene expression. Tumors in which cell cycle gene expression was increased by chemotherapy were likely to be chemotherapy sensitive, whereas tumors in which cell cycle gene transcripts were decreased by chemotherapy were resistant to these agents. A gene expression signature that predicted these changes proved to be a robust and novel index that predicted the response of patients with breast, ovarian, and colon tumors to chemotherapy. Investigations in tumor cell lines supported these findings, and linked treatment induced cell cycle changes with p53 signaling and G1/G0 arrest. Hence, chemotherapy resistance, which can be predicted based on dynamics in cell cycle gene expression, is associated with TP53 integrity.

## INTRODUCTION

Breast cancer cases are increasing and represent a leading cause of cancer death in women [1]. Despite advances in endocrine and targeted therapies, cytotoxic chemotherapies remain mainstays for breast cancer (BC) treatment [2]. However, even potent multi-drug regimens fail to provide long-term cure, especially in the context of advanced disease. For example, combinatorial Doxorubicin (Dx)/Taxotere (Tax) therapy results in complete clinical response in some 20% of cases [3], and provides long-term disease free survival for only ~60% of patients [3, 4]. Accordingly, there is a need for understanding the cellular mechanisms that circumvent chemotherapy efficacy, as well as to identify means to predict which tumors are most likely to respond to therapy.

Many studies focus on identifying gene signatures to guide the selection of appropriate chemotherapy regimens and to identify mechanisms of resistance [5–10]. Such studies generally focus on identifying tumor characteristics or genomic features measured in advance of therapy that correlate with clinical responses. On the other hand, few reports link the dynamics of the measured feature over the course of treatment response. Examples of the latter approach include reports linking endocrine therapy induced reduction of Ki67 labeling as a predictive factor of patient response and outcome [11, 12]. Indeed, changes in Ki67 labeling index measured during treatment is reportedly a superior predictor of response than similar measurements taken prior to treatment [12].

Hence, we investigated neoadjuvant chemotherapy-induced changes of tumor-resident transcripts to identify biological processes that change in response to treatment as these might represent biomarkers that predict therapy response and may reveal mechanisms of treatment resistance.

## RESULTS

### Treatment induced changes of cell cycle gene expression predicts patient response

**Figure 1:**
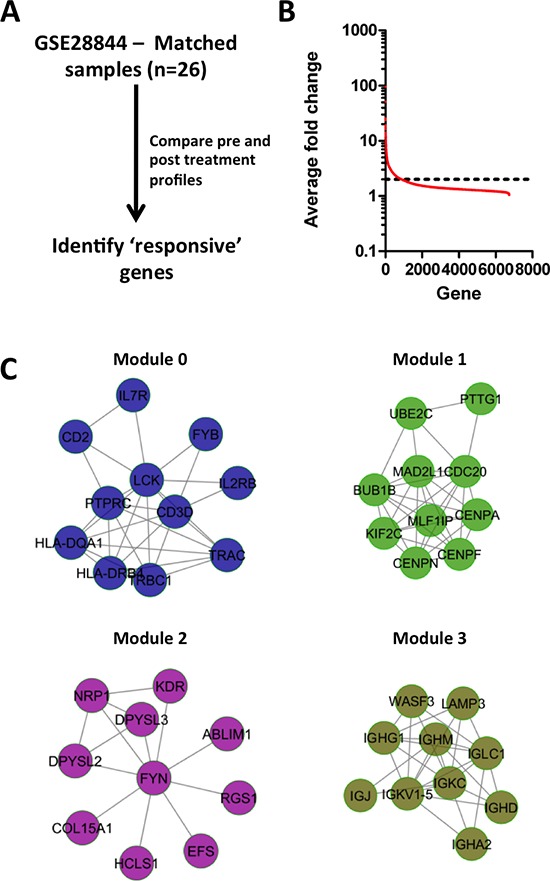
Identification of treatment response genes and modules **(A)** Strategy for identifying treatment responsive genes. **(B)** Average fold change of genes between pre/post treatment biopsies for 26 breast tumors treated with anthracycline & taxane chemotherapy. **(C)** Network modules of genes that display on average at least 2-fold variation between pre/post treatment biopsies.

We sought to identify chemotherapy-induced processes in breast tumors. We reasoned that changes in gene expression resulting from chemotherapy exposure might reveal mechanisms that underpin chemotherapy sensitivity/resistance and identify biomarkers of treatment response. To the latter end we compared published datasets of global gene expression profiles of 26 matched pre/post treatment breast tumor samples from patients that were treated with an anthracycline and taxane (GSE28844 [13]) (Figure 1A), and identified transcripts with at least a 2 fold expression difference between pre/post treatment samples across all 26 tumors (Figure 1B). We mapped these genes as nodes onto a protein interaction network [14, 15], calculated correlation coefficients for all interacting gene pairs, and assigned these as edges to the network [15]. The weighted network was clustered to yield sets of gene interaction modules. Each module comprised sets of genes that are topologically close in a protein interaction network, and also displayed highly coordinate expression among pre/post exposed breast tumor samples. We identified 4 modules individually comprising at least 10 genes with an average correlation that exceeded 0.5 (Figure 1C, Supplementary Table 1).

Independent pathway analyses on each of the modules (Supplementary Table 2) revealed that Modules 0 and 3 were enriched in genes related to T- and B-cell biology, respectively, whereas Module 2 was enriched in genes associated with migratory processes such as axon guidance, and angiogenesis mediated by VEGF and VEGFR. Module 1 was enriched in genes exclusively associated with cell cycle, and included well studied regulators of cell cycle such as the centromere proteins CENPN, CENPF and CENPA that control the separation of sister chromatids during mitosis, as well as BUB1B and CDC20. Notably, proliferative processes have been linked previously with chemotherapy response in breast cancer patients [16–18].

**Figure 2:**
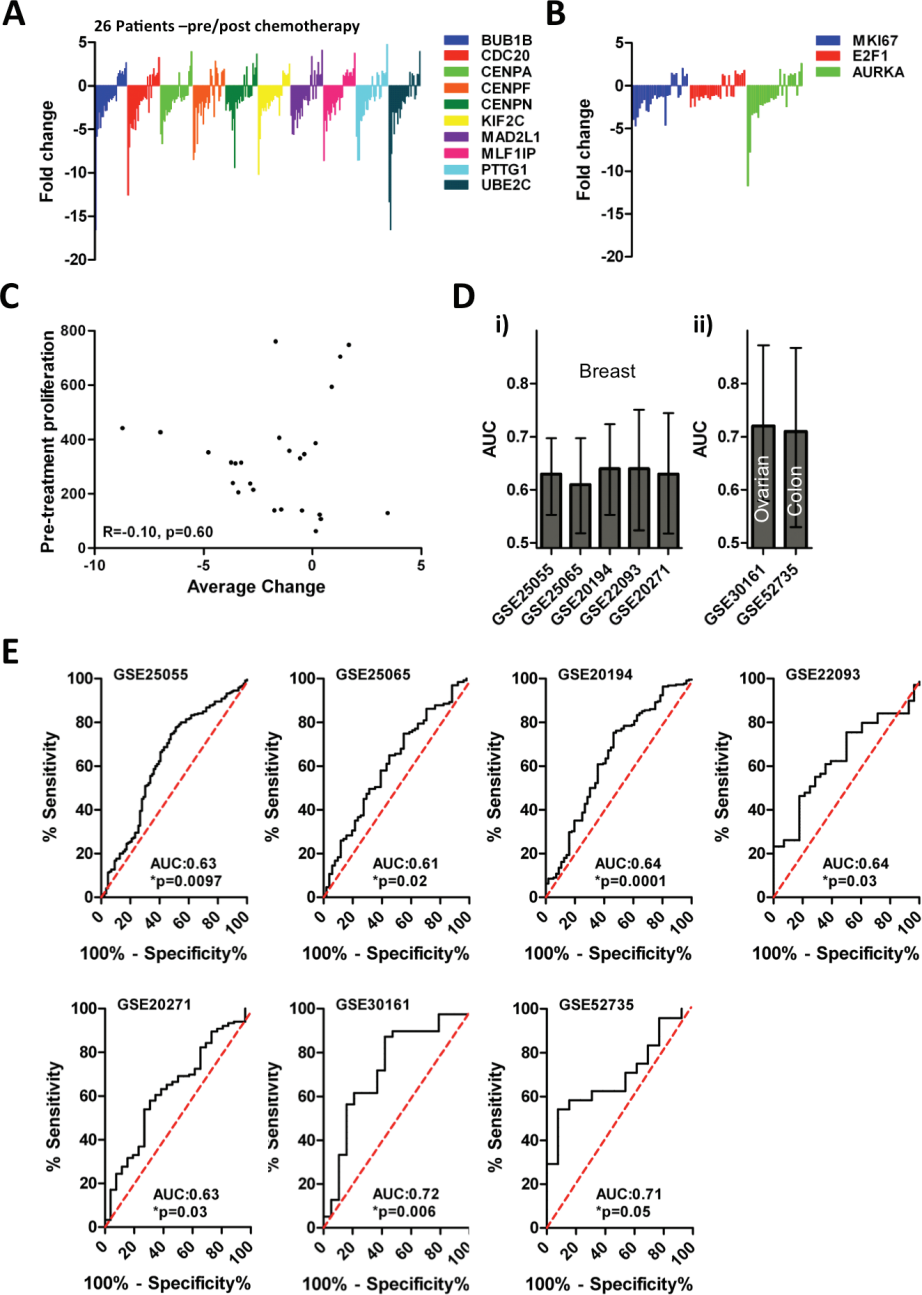
Module 1 gene expression dynamics are associated with therapy response **(A)** Dynamics of module 1 gene expression following therapy is heterogeneous. **(B)** Dynamics of proliferation gene expression following therapy is heterogeneous. **(C)** There is no relationship between Module 1 gene expression prior to therapy and changes in Module 1 gene expression after therapy (*R* = −0.1, *p* = 0.60). **(D)** The RS predicts patient response to chemotherapy among breast cancer (i) as well as ovarian and colon (ii) cancer patients, RS is a significant predictor in each dataset (*p < 0.05, AUC > 0.5). **(E)** ROC analysis of RS in chemotherapy response in 5 breast cancer datasets, one ovarian cancer dataset, and one colon cancer data set.

Despite an established link between the cell cycle and chemotherapy response, the mechanism(s) that governs poor response has yet to be described. Hence we investigated the expression changes of Module 1 genes in the pre/post treatment biopsies. Intriguingly, a third of tumors (*n* = 8) displayed near uniform up-regulation of Module 1 genes in response to chemotherapy treatment (Figure 2A), whereas the remaining two thirds (*n* = 18) showed coordinate down-regulation of Module 1 genes. Additional proliferation associated genes, Ki67, E2F1 and AURKA, that were absent in Module 1, showed similar expression changes among pre/post treatment samples (Figure 2B), strengthening the association of Module 1 with the expression of proliferation-associated genes. These analyses reveal that breast tumors exposed to chemotherapy can be stratified into 2 subsets: 1) tumors that down-regulate cell cycle genes; and 2) tumors that up-regulate cell cycle genes. A comparison of the mean expression level of Module 1 genes and average change in expression levels revealed no correlation between levels of cell cycle gene expression prior to treatment with those found in post treatment tumors (Figure 2C, *r* = −0.1, *p* = 0.60, Spearman's rank correlation). A relationship was also not identifiable between changes in Module 1 during treatment and pre-treatment levels of ki67 transcripts, another well-validated marker proliferation (Supplementary Figure 1A; *r* = –0.14, *p* = 0.47).

We next determined whether changes in Module 1 gene expression during chemotherapy were associated with treatment response. Briefly, we identified a gene signature (Response Signature [RS]) that discriminated between pre-treatment tumors that either up-regulated or down regulated Module 1 genes in response to treatment, and measured the capacity of the RS to predict tumor response to neoadjuvant chemotherapy. To generate the RS, we identified the 10 genes with the largest differential expression between the 6 pre-treatment tumor samples that most highly up-regulated and down-regulated Module 1 gene expression in response to treatment, respectively (Supplementary Table 3). Receiver-operator characteristics curve (ROC) analysis of these 12 patients demonstrated that the RS was significantly associated with whether or not chemotherapy altered Module 1 gene expression in breast tumors (Supplementary Figure 2A, AUC: 1.0, *p* = 0.004). Among the 14 patients that were not used to identify the RS, we validated the capacity of the RS to correctly predict how a tumor would respond to treatment based on changes in Module 1 gene expression (Supplementary Figure 2B, AUC: 0.84, *p = 0.04). Hence, these data demonstrate that the RS can be evaluated on pre-treatment tumor samples and subsequently used to prospectively identify tumors that would up- or down-regulate Module 1 genes in response to chemotherapy. Application of the RS to multiple cohorts of neoadjuvantly treated breast cancer patients revealed a robust relationship between RS and pathological response outcomes for each of the cohorts that we tested (Figure 2D–2E; 5 cohorts; patient *n* = 1066; AUC > 0.5 and *p* < 0.05). Further, the predictive nature of the RS could also identify response to chemotherapy in colon and ovarian patient cohorts (Figure 2D–2E; Ovarian: *n* = 58, Colon: *n* = 37; AUC > 0.5 and *p* < 0.05). In each cohort, higher signature scores were significantly associated with resistance to chemotherapy (Supplementary Figure 2C), strongly suggesting that the treatment-induced down-regulation of Module 1 genes is also associated with treatment resistance.

A final analysis was conducted to investigate the prognostic capacity of the RS while accounting for clinical factors, by performing multivariate regression analyses in a pooled breast cancer cohort, made by combining the 5 neoadjuvant breast cohorts presented above. Although the pooled cohort comprised 1066 patients, only 895 patients had complete clinical annotations including RS score, grade, age, as well as ER and node status (summarized in Supplementary Table 4). Univariate analysis revealed that the RS signature (OR: 2.95 [2.14–4.08], *p* < 0.001), ER status (OR: 0.20 [0.15–0.29], *p* < 0.001), and grade (OR: 4.36 [3.00–6.34], *p* < 0.001) were all significant prognosticators of response, whereas age and node status were not. In the multivariate model, the RS signature ( *p* = 0.032), ER status ( *p* < 0.001) and grade ( *p* < 0.001) all remained significantly associated with response (Supplementary Table 5). Hence these data confirm that the RS signature is a significant prognosticator of response even after adjusting for standard clinical variables. Together, these data suggest that exposure of breast, ovarian or colorectal tumors to chemotherapy leads to altered cell cycle gene expression. Decreased expression of proliferation genes following patient treatment is associated with chemotherapy resistance, whereas increased or no change in the expression of proliferation genes after chemotherapy predicts sensitivity to such agents.

### Cell cycle integrity governs breast cancer cell lines response to chemotherapy

**Figure 3:**
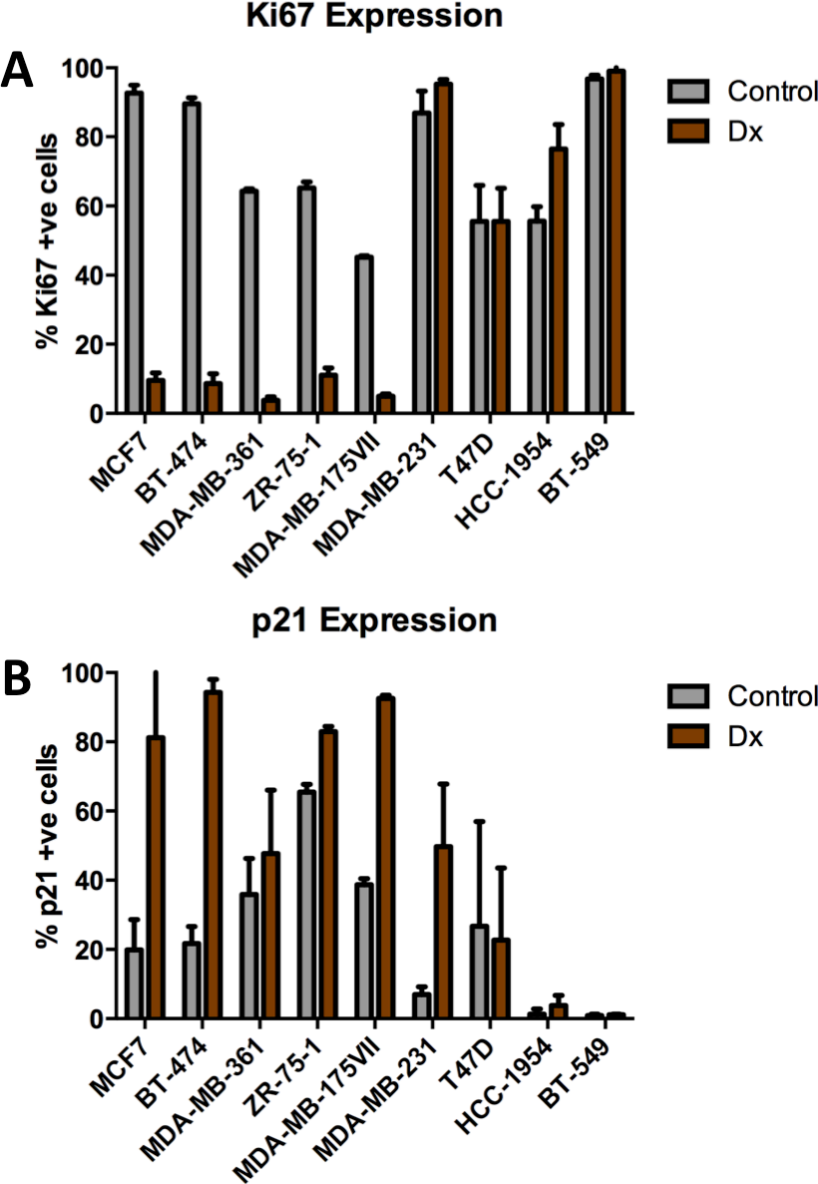
Effects of chemotherapy on Ki67 and p21 expression in breast tumor cell lines **(A)** Ki67 is heterogeneously expressed in breast tumor cell lines in response to doxorubicin (100 nM). **(B)** p21 is variably expressed among breast tumor cell lines following treatment with doxorubicin (100 nM).

To establish a mechanism(s) that links altered proliferation gene expression and chemotherapy resistance, we treated 9 different breast cancer cell lines with Dx, an anthracycline (100 nM for 2 days), and measured cell cycle response by staining for markers of cell proliferation (Ki67) and cell cycle arrest (p21). The cell lines displayed 2 distinct behaviors: those in which Ki67 expression was decreased (MCF7, BT474, MDA-MB-361, ZR-75-1 and MDA-MB-175VII), and those in which Ki67 was increased or unchanged (T47D, MDA-MB-231, HCC1954 and BT-549) (Figure 3A). Staining for p21 typically demonstrated the converse trend (Figure 3B). Moreover, re-analysis of published microarray based gene expression profiling of control and Dx treated MCF7 cells [19], revealed near uniform (8/10) down-regulation of Module 1 genes in response to Dx, further supporting the capacity of cell lines to model our previous observations made in breast tumors (Supplementary Figure 3).

**Figure 4:**
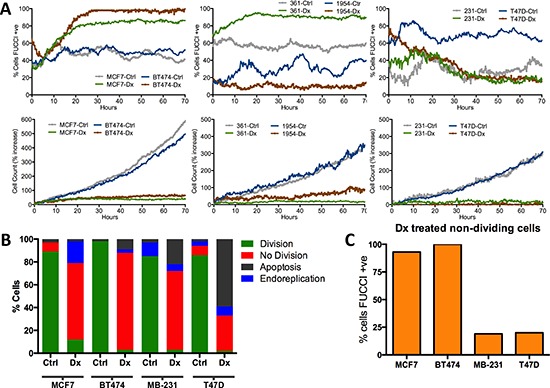
The cell cycle response of human breast cancer cell lines to doxorubicin treatment **(A)** Percentage of cells expressing FUCCI-G1 and cell count increase over 3 days in the presence of doxorubicin (Dx) or control (Ctrl) conditions. **(B & C)** Summary of single cell tracking of human BC lines in control or doxorubicin conditions. B) Chart depicts the percentage of cells for each line and condition in different stages of the cell cycle. C) Percentage of non dividing doxorubicin treated cells that are expressing FUCCI-G1 reporter.

We extended these findings using the H2BGFP-FUCCI cell cycle reporter [20, 21] that encodes a fusion of H2B-GFP, which decorates chromatin, and mKO2-Cdt1 (herein referred to as FUCCI-G1) [21], the expression of which is restricted to G1/G0. Live cell imaging over the course of 3 days following Dx treatment showed that, although all cell lines displayed a marked decrease in proliferation, nearly all MCF7, BT474 and MDA-MB-361 cells increased or maintained expression of the FUCCI-G1 reporter 24 hours after treatment, whereas the fraction of T47D, MDA-MB-231 or HCC1945 cells expressing FUCCI-G1 remained constant or decreased (Figure 4A & Supplementary Figures 4A–4F). Importantly, the results were consistent across a range of Dx doses, including previously defined IC50 doses for each line (Supplementary Figure 5A) (10). Single cell tracking of the reporter cell lines revealed that Dx treated MCF7 and BT474 cells yielded non-dividing cells that were nearly all arrested in the G1/G0 phase of the cell cycle (Figures 4B–4C & Supplementary Figure 5B). In contrast, Dx treatment of T47D and MDA-MB-231 cells lead to increased apoptosis, and non-dividing cells that did not express FUCCI-G1 (Figures 4B–4C & Supplementary Figure 5B). In addition to using the FUCCI-G1 reporter, we also employed BrDU staining to monitor cell cycle status. BrDU incorporation in Dx treatment of MCF7 and MDA-MD-231 cells confirmed our observation that Dx induced MCF7 G1 arrest, whereas MDA-MB-231 cells arrested primarily in G2/M (Supplementary Figure 5C). In sum, some breast cancer cell lines (MCF7, BT474, MDA-MB-361, ZR-75-1 and MDA-MB-175VII) phenocopied tumors that down-regulated cell cycle gene expression in response to treatment, whereas other lines (T47D, MDA-MB-231, HCC1954 and BT-549) were similar to those that increase cell cycle gene expression after treatment.

To identify the mechanism(s) that regulate chemotherapy-induced cell cycle gene changes, we performed gene set enrichment analysis (GSEA) with publically available gene expression profiles of Dx treated MCF7 cells, which identified enrichment for multiple p53 related gene sets (Supplementary Table 6; bolded gene sets) [22]. The latter is in keeping with the fact that MCF7 cells harbor WTp53 alleles [23, 24], and is consistent with the fact that Dx treatment of these cells activated G1/G0 arrest via functional p53 signaling.

We hypothesized that the two distinct cell cycle response patterns observed in our panel of H2BGFP-FUCCI expressing cell lines might be related to their *TP53* status, as p53 mediates G1/G0 cell cycle arrest in response to DNA damage [25, 26]. We performed time-lapse imaging of H2BGFP-FUCCI cell lines grown in the absence or presence of the small molecule Nutlin3A, an inhibitor of the interaction of p53 with MDM2. MDM2 targets p53 for degradation and hence Nutlin3A activates p53 signaling in cell lines that harbor the WTp53 alleles. Nutlin3A elicited growth arrest and FUCCI-G1 expression or Ki67 loss in the same lines (MCF7, BT474, MDA-MB-361, ZR-75-1 and MDA-MB-175VII) that were arrested in G1/G0 in response to Dx exposure (Supplementary Figures 6A–6D). However, Nutlin3A, like Dx, did not effect G1/G0 cell cycle arrest in the T47D, MDA-MB-231, HCC1945 or BT-549 cell lines, which harbor mutant *TP53* alleles. These results were further confirmed by BrDU incorporation analysis in MCF7 and MDA-MB-231 cells (Supplementary Figure 5C). Taken together these findings suggest that down-regulation of cell cycle gene expression is dependent on functional p53 signaling, ultimately leading to chemotherapy resistance.

### Functional p53 signaling is associated with chemotherapy resistance in breast cancer patients

**Figure 5:**
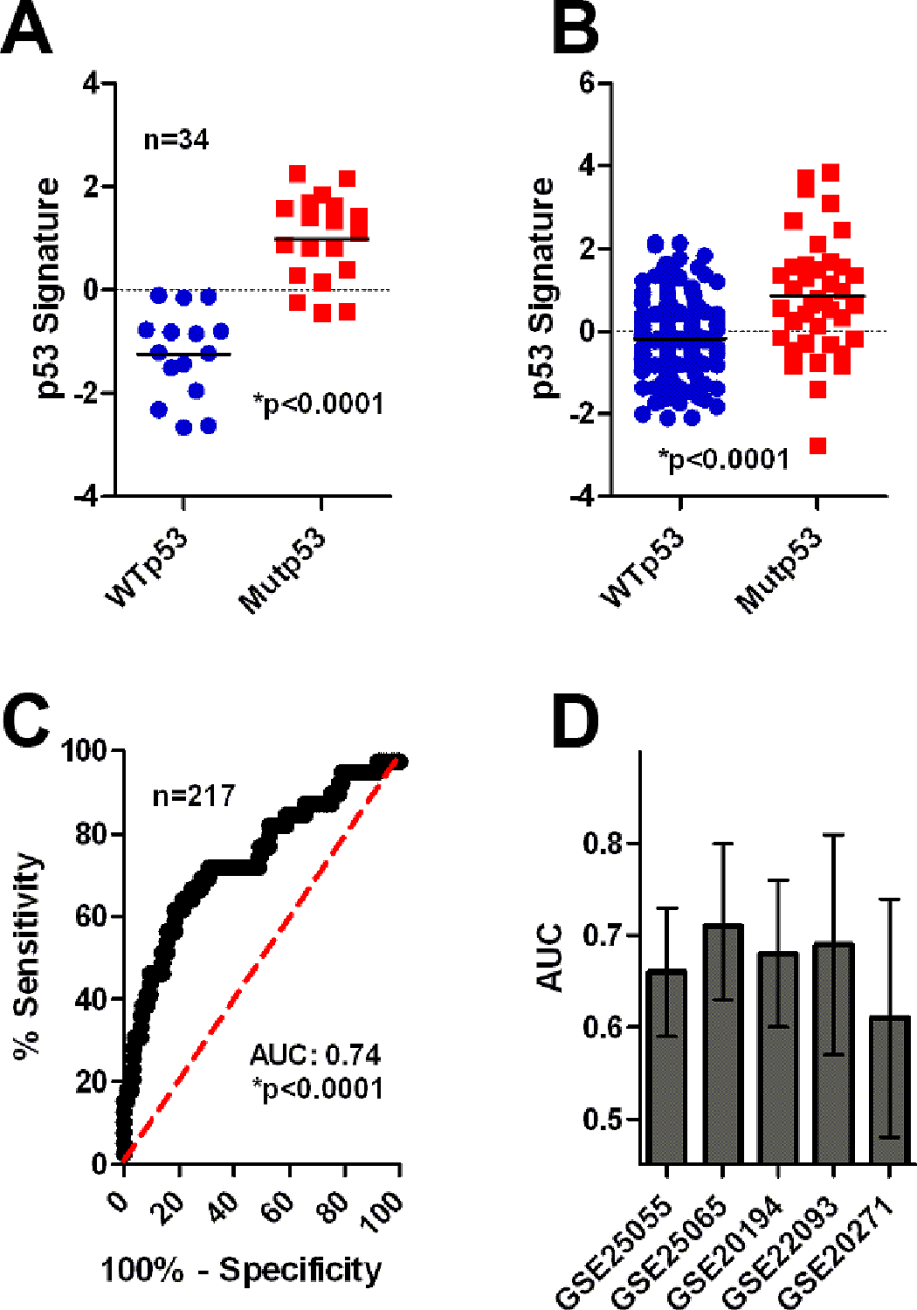
Mutp53 is associated with chemotherapy sensitivity in breast cancer patients **(A)** The p53 signature correctly identifies MUTp53/WTp53 tumors in the training cohort. **(B)** The p53 signature correctly identifies MUTp53/WTp53 tumors in the validation cohort by *t*-test analysis (*p < 0.0001). **(C)** The p53 signature correctly identifies MUTp53/WTp53 tumors in the validation cohort by ROC analysis (*p < 0.0001). **(D)** The p53 signature is associated with patient response in 5 neoadjuvant cohorts of breast cancer patients (AUC and 95% confidence interval is shown).

Our data described above suggested that a reduction of cell cycle gene expression following therapy is associated with chemotherapy resistance and that this is dependent, at least in part, by activation of functional p53 signaling. Based on this data we hypothesized that intact p53 signaling in human breast cancer patients would likely be associated with chemotherapy resistance. To confirm this hypothesis, we sought to test whether a transcriptional signature of *TP53* mutational status was associated with response to neoadjuvant chemotherapy, similar to our observations made with the RS. We first interrogated transcriptional data (GSE3494) of 251 breast tumors for which the mutational status of *TP53* was also known [27]. In short, we used PAM and 10-fold cross-validation to identify an 18 probe set signature that was associated with p53 status in patient samples (*n* = 34, training cohort) comprising a subset of the original GSE3494 cohort (Supplementary Table 7). Expectedly, we found that among training patients, those whose tumor harbored mutant *TP53* had significantly higher p53 signature scores than those with WT *TP53* (Figure 5A, *t*-test, *p < 0.0001). Among the remaining patients in the GSE3494 cohort (*n* = 217, validation cohort) we validated the capacity of the p53 gene signature to predict *TP53* status, and found that the p53 signature remained significantly associated with tumor p53 status using both *t*-tests and receiver operator characteristic (ROC) curve analysis (Figures 5B–5C, *t*-test, *p < 0.0001, AUC: 0.74, *p < 0.0001). Accordingly, these data suggest that our p53 signature can be used as a surrogate marker of tumor *TP53* status, and represents a useful tool to examine *TP*53 status in additional transcriptional breast tumor datasets.

We next measured the relationship between tumor *TP*53 status and response to neoadjuvant chemotherapy. Application of the p53 signature in the same 5 cohorts of neadjuvant treated breast cancer patients revealed a robust relationship between the p53 signature and patient response (Figure 5D & Supplementary Figure 7, Supplementary Table 8; 5 cohorts; total *n* = 1066; AUC > 0.5). In 4 of 5 cohorts the AUC was significantly above 0.5, whereas the 5^th^ cohort (GSE20271) narrowly missed significance (*p* = 0.07). In all cases elevated p53 signature scores, indicating mutant *TP53* status, was associated with chemotherapy sensitivity. Accordingly, we concluded that mutant *TP*53 status, as assessed by our transcript signature, was associated with chemotherapy sensitivity.

### Induction of G1 arrest by p53 results in chemotherapy resistance

Our data suggested that activation of p53 signaling by either chemotherapy induced DNA damage or small molecules that prevent p53 degradation decreased cell cycle gene expression resulting in G1/G0 arrest in a subset of breast tumor cell lines harboring WT *TP53* alleles. Moreover, analysis of clinical data suggested this mechanism might drive therapy resistance whereby cells avoid cell death during exposure to cytotoxic agents.

**Figure 6:**
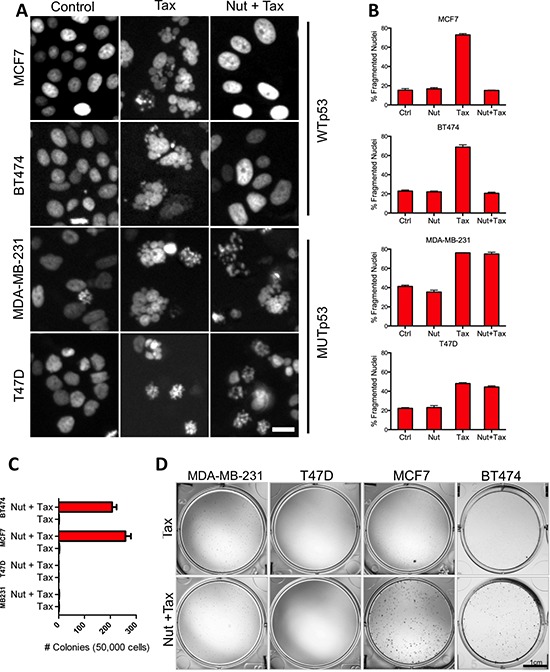
Activation of functional p53 signaling confers chemotherapy resistance to breast tumor cells **(A)** Representative day 3 micrographs of WTp53 (MCF7 & BT474) and MUTp53 (MB-231 & T47D) cell response to Tax only or combined Nutlin 3A and Tax treatment. Scale bar = 20 um. **(B)** Graphs comparing the percentage of Tax-induced nuclear fragmentation at day 3 in the absence or presence of Nutlin 3A. **(C)** Images of wells stained for colonies in MCF7 and 231 plates (Scale bar = 1 cm). **(D)** Colony forming efficiency of cell lines following Tax only or combined Nutlin 3A and Tax treatment.

To test this hypothesis directly we first examined cell nuclear morphology in response to Tax, a chemotherapeutic agent that stabilizes microtubules thus leading to mitotic catastrophe and nuclear fragmentation [28]. We exposed cells to either Tax (100 nM) alone or a combination of Nutlin3A (10 μM) and Tax, and then compared the frequency of fragmented nuclei among cell lines with WT *TP53* (MCF7 & BT474) or mutant *TP53* (MDA-MB-231 & T47D). Irrespective of the cell line, exposure of breast tumor cells to Tax alone rapidly induced nuclear fragmentation in ~70% of nuclei (Figures 6A–6B). G1/G0 arrest of the cell lines harboring WT *TP53* with Nutlin3A during exposure to Tax rescued the lines from Tax-induced nuclear fragmentation, (Figure 6A). Importantly, Nutlin3A did not protect cell lines harboring mutant *TP53* from Tax-induced nuclear fragmentation (Figures 6A–6B).

We performed colony-forming assays with cells that had been exposed to Tax or Tax and Nutlin3A to establish whether division-competent viable cells remained after exposure to these agents. Cells were either incubated with vehicle (control) or Nutlin3A for 3 days, with both receiving a 24 hour treatment of Tax at the beginning of day 2. While vehicle controls grew to confluency (data not shown), Tax-only treatment in the presence of vehicle inhibited the capacity of both WT *TP53* and mutant *TP53* cells to form colonies (Figures 6C–6D). However, the presence of Nutlin3A during Tax treatment protected the capacity of cells with WT *TP53* to form colonies, but not those with mutant *TP53*. These data show that activation of functional p53 signaling and G1/G0 arrest protects cells during exposure to the cytotoxic effects of Tax.

## DISCUSSION

Defining the currently elusive mechanisms that underlie cancer patient response to chemotherapy will yield important advances in patient care, including redirecting non-responders to alternative treatment strategies that may afford better outcomes [29].

Here we describe the novel observation that chemotherapy induced cell cycle gene expression changes is predictive of patient response. To date, most reports relating patterns in tumor gene expression to clinical responses have focused primarily on discovering genomic features in pre-treatment biopsies that predict therapy response [5–10]. However, we report that changes in gene expression occurring during response to chemotherapy are also related to patient response. We identified a set of 10 genes associated with cellular proliferation, termed Module 1, that are among the most variably expressed genes within tumors following treatment. Module 1 genes are coordinately regulated intra-tumorally, and are uniformly up or down-regulated in response to chemotherapy. RS, a surrogate measure for changes in Module 1 gene expression, was significantly related to patient response in 5 cohorts of breast cancer patients (total *n* = 1066), as well as in smaller cohorts of colon and ovarian cancer patients (*n* = 37 and *n* = 58, respectively). Specifically, tumors predicted to increase Module 1 gene expression following chemotherapy were more likely to respond favorably to treatment, whereas the opposite was true for tumors predicted to reduce Module 1 gene expression in response to chemotherapy.

From a clinical standpoint, pre-treatment evaluation of the RS or monitoring cell cycle changes during the course of neoadjuvant chemotherapy, might represent valuable predictive biomarkers. In this fashion, patients identified as resistant to chemotherapy could benefit from expedient enrollment in clinical trials investigating the efficacy of novel agents [29]. However, many issues remain to be addressed to confirm the clinical utility of either the RS or monitoring Module 1 genes during treatment. For example, our conclusions are based on the analysis of retrospective data, which limits its clinical value, and our analyses of colon and ovarian tumors were limited by the small sample sizes available. Prospective clinical trials would establish a full understanding of the utility of the approach presented here.

To date, most predictive gene signatures identified for breast cancer derive their predictive capacity based on their ability to measure proliferation in pre-treatment tumor samples [6, 9, 17]. The relationship between proliferation and response is further supported by reports that ki67 staining is also a robust predictor of chemotherapy response [30–32]. In the initially discovery dataset (GSE28844) we did not observe a significant relationship between the RS and Ki67, although some caution is warranted when interpreting this result as it is only based on analysis of 26 samples. Nonetheless, these data overall suggest that the RS may capture distinct predictive information from either Ki67 staining or purely proliferation based signatures.

The success of the RS to identify chemotherapy responsive tumors led us to investigate the phenomenon whereby therapy response occurs. To this end we used breast tumor cell lines to discover that a subset of cell lines examined entered G1/G0 cell cycle arrest after exposure to Dx. Bioinformatic and *in vitro* experiments linked this behavior with *TP53* integrity: activation of p53 signaling was sufficient to induce G1/G0 arrest, which in turn protected cells from further chemotherapy treatments, thus permitting subsequent tumor cell regrowth post-therapy. Many clinical studies have examined the relationship between *TP53* status and drug response: some were inconclusive [33, 34], whereas others linked chemotherapy responsiveness/sensitivity with either WT *TP53* [35–37] or mutant *TP53* [38–40]. Our study found that *TP53* integrity is associated with chemotherapy outcome, and that WT *TP53* is most strongly connected with resistance to the cytotoxic effects of chemotherapy. Indeed, MMTV-*Wnt1* mouse mammary tumors with mutant *TP53* are significantly more sensitive to Dx than those with WT *TP53* [41]. The composition of the RS is intriguing, as it includes the gene WRAP53 (WDR79). WRAP53 is a naturally occurring *TP53* antisense transcript that has been demonstrated to positively regulate p53 levels via RNA interaction [42]. Based on the RS, WRAP53 expression is higher in tumors that respond to chemotherapy through reduced expression of cell cycle genes, consistent with its role as a positive regulator of p53 and its association with resistance.

Intriguingly, although *TP53* mutations are known to occur in a molecular subtype specific fashion among breast cancer patients [43, 44], no study has examined the relationship between *TP53* status and chemotherapy response in a subtype specific fashion. Taken with this data, our results provide a possible explanation for the differences in chemotherapeutic sensitivity observed between breast tumors of differing molecular subtypes [7, 45, 46]. For example, basal-like breast tumors harbor the highest frequency of mutations in *TP53* [43, 47] and patients with basal-like tumors are known to experience the highest rates of pathological response. Conversely, luminal breast tumors harbor the lowest frequency of mutations in *TP53* and patients with luminal tumors experience the lowest rates of pathological response. It will be interestingly to learn if mutations in *TP53* are associated with chemotherapy sensitivity in other tumor types. Indeed, a recent report suggests that mutations in *TP53* are associated with favorable responses to chemotherapy in ovarian cancers [48].

One important aspect to consider when interpreting the data we present here is the role of therapy-induced senescence (TIS). TIS is a common tumor response to cytotoxic chemotherapies, including Dx [49], which results in permanent growth arrest, via halting the cell cycle in G2/M or G1 phase [50]. TIS shares overlapping characteristics with normal physiological senescence, including several genetic pathways that have been implicated in TIS, such as the tumour suppressors p53 and Rb. However it is important to note that WT *TP53* and mutant *TP53* cells [49], as well as cells completely lacking *TP53* [51, 52] can undergo TIS, indicating that functional p53 signalling is not a required component for attaining TIS.

The extent to which TIS defines a patient's response to chemotherapy is not fully understood, but it is clear that TIS is not observed in all tumor cells, and that a small fraction of cells can evade TIS and reinitiate growth [52]. A key experiment presented here demonstrated that activation of a functional p53 program in response to nutlin3A was protective against the cytotoxic effects of chemotherapy (Figures 6A–6D), a result that is consistent with previous important studies linking p53 induction and mitotic inhibitor protection [53–60]. Although this observation is not necessarily consistent with p53 mediated induction of senescence, as treated cells retained the capacity to proliferate and make colonies. Hence, p53 driven resistance likely also includes a non-senescent mechanism(s) of cell cycle arrest. However, the transcriptional analyses presented here may generally afford a new avenue for investigating TIS mechanisms of patient response to chemotherapy.

Overall, the findings we present here have several important implications. Clinically, they suggest that either the RS signature or directly monitoring changes in cell cycle gene expression during treatment could be used for timely identification of non-responders, who could then be rapidly switched to alternate therapeutic options. Moreover, our findings also link *TP53* integrity with treatment-induced changes in tumor cell responsiveness to chemotherapeutic drugs. Hence, modulation of p53 signaling with various small molecules depending on pathway integrity could serve to increase potency of commonly used chemotherapeutic agents. Finally, we extended our finding in ovarian and colon tumors, suggesting that the implications of our work extend beyond breast tumors and may apply to the majority of tumors independent of tissue-of-origin.

## METHODS

### Bioinformatics

Full details of the Bioinformatics methods can be found in the Supplementary Methods.

### Cell culture & generation of H2GFOIP reporter lines

All cell lines were obtained from the ATCC, characterized by STR profiling, and passaged minimally prior to completing these experiments. MCF7, BT-474, MB-MDA-231, T47D, HCC-1954, ZR-75-1, MDA-MB-175VIII, BT549, and MDA-MB-361 BC cell lines were cultured as previously described [23]. Stable clones (MCF7, HCC1954, and MD-MBA-231) expressing the H2GFOIP reporter were produced by electroporation (300 v, 250 uF, 0.4 mm cuvette gap) with pCAG H2GFOIP plasmid DNA [20] and selected by the addition of 1 ug/ml puromycin to the medium. Other breast cancer reporter lines that proved refractory to electroporation-mediated transgenesis (BT-474, T47D, and MDA-MB-361) were infected with a H2GFO lentivirus. The recombinant H2GFO lentivirus was constructed as follows. The H2BGFP-F2A-mKO2-Cdt1 fragment was released from pCAG-H2GFOIP by digestion with KpnI and PmeI, and then subcloned into KpnI/EcoRV cut pEF-1a/pENTR (Addgene #17427) [61]. A LR clonase (Invitrogen) recombination reaction was then used to deliver pEF-1a H2BGFP-F2A-mKO2-Cdt1 into the pLenti X1 puro DEST vector (Addgene #17297). Virus was prepared as previously described, and stably infected cells were selected by the addition of 1 ug/ml puromycin to the medium.

### Immunocytochemistry and flow cytometry

Immunocytochemistry was performed in 96 well plates. Cells were fixed and stained as previously described [62] with antibodies to p21 (rabbit polyclonal, Cell Signaling # 2947; 1:400 dilution) or Ki67 (mouse monoclonal, Santa Cruz # SC23900; 1:100 dilution) and with appropriate secondary AF647 antibodies (Invitrogen #A-21238 & #A-31571; 1:500 dilution). Nuclei were co-stained with Hoechst 33342. BrdU incorporation experiments were performed as per manufacturers instructions (BD FITC BrdU Flow Kit #559619) and acquired on a LSRII (BD). Analysis was performed using FlowJo software.

### Live cell imaging and high content imaging

Details for live and high content imaging are described previously [20, 62]. Briefly, for live cell imaging, cell lines were seeded in 12 well plates at 50,000 cells per well, and imaged using a Biostation CT live imaging system (Nikon). Dx or Nutlin3A was added immediately prior to imaging, and images were acquired every 15 minutes for 3–4 days, compiled in ImageJ and then analysed with custom ImageJ scripts. Cell tracking was performed manually in ImageJ using the MtrackJ plugin or semi-automated with custom software written in Matlab 2012a (MathWorks, Natick, MA). A cross-correlation algorithm for automated cell tracking was implemented on a graphics workstation (Intel Xeon E5–1607, 16 GB RAM, equipped with a Tesla C2075 GPU). The Matlab cell tracking platform generates division pedigree databases. The latter were interrogated to yield individual cell features such as FUCCI-G1 fluorescence, G1 and S-phase duration and annotated fates (division, apoptosis, endoreplication, lost, or end of experiment). The database platform includes tools for display of single cell data such as heat maps showing single cell FUCCI-G1 expression dynamics as bar graphs or division trees. For high content imaging, cells were seeded at 10,000 cells per well of 96 well plates and imaged using an Operetta High Content Imaging system (Perkin Elmer). High content imaging data was transferred to a Columbus (Perkin Elmer) database and ~1,000–2,000 cells per n were analyzed with custom scripts in Acapella (Perkin Elmer) that segmented nuclear objects by Hoechst or H2BGFP, and subsequent intensity quantification for FUCCI-G1 or AF647 signals were performed. Fragmentation analysis was performed using Acapella-based phenoLOGIC machine learning after supervised identification of normal and TAX-induced fragmented nuclei.

### Statistical analysis

The predictive capacity of the RS signature was evaluated using receiver-operator characteristic curve (ROC) analysis, as well as univariate and multivariate logistic regression. *T*-tests were also used to compare RS signature scores between responsive and non-responsive tumors. All tests were two-sided and a *p*-value of 0.05 or less was considered statistically significant. All graphs for *in vitro* experiments show standard deviation derived from *n* = 3–5.

## SUPPLEMENTARY METHODS


