# Supplementary material for: Treatment-induced cell cycle kinetics dictate tumor response to chemotherapy
Source: Oncotarget. 2015 Feb 7;6(9):7040–52. doi: 10.18632/oncotarget.3140 (PMC4466668; doi:10.18632/oncotarget.3140)
Supplement: Supplementary file 1 [file oncotarget-06-7040-s001.pdf]

SUPPLEMENTARY METHODS

Bioinformatics

Patients and samples

All data was publicly available and downloaded from the gene expression omnibus (http://www.ncbi.nlm.nih.gov/geo/). The raw intensity files (.CEL) comprising each dataset were download and normalized using the Robust Multichip Algorithm (RMA) to generate probe set intensities (1). The following datasets were used in this study (GSE28844, GSE19368, GSE20194, GSE25055, GSE25065, GSE22093, GSE20271, GSE30161, GSE52735).

Pre and post treatment analysis

We downloaded gene expression data for 61 pre/post anthracycline & taxane based treatment tumor biopsies (GSE28844). Together these samples represent matched tumor biopsies (n = 28), as well as 4 pre-treatment and 1 post-treatment tumor biopsies. As 2 matched samples experienced a complete response (no residual tumor profiled in post-chemotherapy samples), we completed our analysis with the remaining 26 matched samples.

Network analysis

All network analysis was carried out using Cytoscape (v2.8.2) and the Reactome FIs plugin(2).

Response signature

The Response signature [RS] identified using patients from cohort GSE28844 that maximally changed cell cycle genes in response to treatment. We selected the top 10 genes that were maximally differentially expressed (lowest p-value, t-test), between the 6 tumors that most and least up-regulated cell cycle gene expression in response to therapy.

P53 gene signature

The P53 gene signature was identified using the GSE3494 patient cohort, which comprised 251 patients for whom the mutational status of the *TP53* gene is known (7). Briefly, we identified an optimized p53 signature with a sub-cohort of 34 patients using Prediction Analysis of Microarrays (PAM)and 10-fold cross-validation (8). The remaining 217 patients were used to validate the capacity of the p53 signature to discriminate between tumors with either WTp53 or MUTp53.

Chemotherapy response

Multiple cohorts were studied to test whether the RS was related to sensitivity or resistance to chemotherapy (Breast: GSE20194, GSE25055, GSE25065, GSE22093, GSE20271, Ovarian: GSE30161, Colon: GSE52735, Supplementary Table 8). For breast, tumors that did not achieve a complete pathological response (pCR) during treatment were considered to be chemotherapy resistant. For ovarian, tumors that progressed within 18 months of treatment were considered to be chemotherapy resistant. For colon, tumors that did not respond to chemotherapy were considered to be chemotherapy resistant. The response characteristics of these datasets are summarized in Supplementary Table 3. To evaluate the RS/p53 signature, the expression values for each probe set were transformed such that the mean and standard deviation were set to 0 and 1 in each dataset, respectively. A signature score was calculated for each patient as follows (3, 4).

$$\frac{\sum_{i \in P} x_i}{n_P} - \frac{\sum_{i \in N} x_j}{n_N}$$

Where x is the transformed expression, n is the number of probe sets, P is the set of probes with reported positive correlation to tumors that up-regulate cell cycle genes in response to treatment/have mutTP53, and N is the set of probes with reported negative correlation to tumors that down-regulated cell cycle genes in response to treatment/have wtTP53. Finally, all signature score values were standardized within each of the tested datasets.

Gene set enrichment analysis

Gene Set Enrichment Analysis (GSEA) was performed using the gene expression profiles of Dx treated and vehicle treated MCF7 cells (GSE18552)(5) as previously described, using gene set permutation to estimate False Discovery Rate (FDR)(6).

REFERENCES

1. Irizarry RA, Hobbs B, Collin F, et al. Exploration, normalization, and summaries of high density oligonucleotide array probe level data. Biostatistics. 2003; 4:249–264.
2. Wu G, Feng X, Stein L. A human functional protein interaction network and its application to cancer data analysis. Genome Biol. 2010; 11:R53.
3. Hallett RM, Dvorkin-Gheva A, Bane A, Hassell JA. A Gene Signature for Predicting Outcome in Patients with Basal-like Breast Cancer. Sci Rep. 2012; 2:227.
4. Hallett RM, Pond G, Hassell JA. A target based approach identifies genomic predictors of breast cancer patient response to chemotherapy. BMC Med Genomics. 2012; 5:16.

5. Iorio F, Bosotti R, Scacheri E, et al. Discovery of drug mode of action and drug repositioning from transcriptional responses. Proc Natl Acad Sci U S A. 107:14621–14626.
6. Subramanian A, Tamayo P, Mootha VK, et al. Gene set enrichment analysis: a knowledge-based approach for interpreting genome-wide expression profiles. Proc Natl Acad Sci U S A. 2005; 102:15545–15550.
7. Miller LD, Smeds J, George J, et al. An expression signature for p53 status in human breast cancer predicts mutation status, transcriptional effects, and patient survival. Proc Natl Acad Sci U S A. 2005; 102:13550–13555.
8. Tibshirani R, Hastie T, Narasimhan B, Chu G. Diagnosis of multiple cancer types by shrunken centroids of gene expression. Proc Natl Acad Sci U S A. 2002; 99:6567–6572.

SUPPLEMENTARY TABLES AND FIGURES

Supplementary Table 1: Module genes

Module	Gene	Description
0	CD2	CD2 molecule /// CD2 molecule
0	CD3D	CD3d molecule, delta (CD3-TCR complex)
0	FYB	FYN binding protein (FYB-120/130)
0	HLA-DQA1	major histocompatibility complex, class II, DQ alpha 1
0	HLA-DRB4	major histocompatibility complex, class II, DR beta 4
0	IL2RB	interleukin 2 receptor, beta /// interleukin 2 receptor, beta
0	IL7R	interleukin 7 receptor /// interleukin 7 receptor
0	LCK	lymphocyte-specific protein tyrosine kinase
0	PTPRC	protein tyrosine phosphatase, receptor type, C
0	TRAC	T cell receptor alpha constant /// T cell receptor alpha constant
0	TRBC1	T cell receptor beta constant 1
1	BUB1B	BUB1 budding uninhibited by benzimidazoles 1 homolog beta (yeast)
1	CDC20	CDC20 cell division cycle 20 homolog (S. cerevisiae)
1	CENPA	centromere protein A, 17 kDa
1	CENPF	centromere protein F, 350/400 ka (mitosin) /// centromere protein F, 350/400 ka (mitosin)
1	CENPN	centromere protein N
1	KIF2C	kinesin family member 2C
1	MAD2L1	MAD2 mitotic arrest deficient-like 1 (yeast)
1	MLF1IP	MLF1 interacting protein
1	PTTG1	pituitary tumor-transforming 1
1	UBE2C	ubiquitin-conjugating enzyme E2C
2	ABLIM1	actin binding LIM protein 1
2	COL15A1	collagen, type XV, alpha 1
2	DPYSL2	dihydropyrimidinase-like 2
2	DPYSL3	Dihydropyrimidinase-like 3
2	EFS	embryonal Fyn-associated substrate
2	FYN	FYN oncogene related to SRC, FGR, YES
2	HCLS1	hematopoietic cell-specific Lyn substrate 1
2	KDR	kinase insert domain receptor (a type III receptor tyrosine kinase)
2	NRP1	Neuropilin 1
2	RGS1	regulator of G-protein signalling 1
3	IGHA2	Immunoglobulin heavy constant alpha 2
3	IGHD	immunoglobulin heavy constant delta
3	IGHG1	Immunoglobulin heavy constant gamma 1 (G1m marker)
3	IGHM	immunoglobulin heavy constant mu

(*Continued*)

Module	Gene	Description
3	IGJ	Immunoglobulin J polypeptide, linker protein for immunoglobulin alpha and mu polypeptides
3	IGKC	Immunoglobulin kappa constant
3	IGKV1–5	Immunoglobulin kappa variable 1–5
3	IGLC1	immunoglobulin lambda constant 1 (Mcg marker)
3	LAMP3	lysosomal-associated membrane protein 3
3	WASF3	WAS protein family, member 3

Supplementary Table 2: Pathway analysis of modules

Module	Pathway	FDR
0	Cell adhesion molecules (CAMs)(K)	1.25E-04
0	HTLV-I infection(K)	1.43E-04
0	Hematopoietic cell lineage(K)	1.67E-04
0	T cell activation(P)	2.00E-04
0	TCR signaling in naive CD8+ T cells(N)	2.22E-04
1	Cell cycle(K)	2.50E-04
1	Cell Cycle Checkpoints(R)	3.33E-04
1	Regulation of mitotic cell cycle(R)	5.00E-04
1	PLK1 signaling events(N)	8.00E-04
1	Mitotic M-M/G1 phases(R)	1.00E-03
2	Axon guidance(K)	5.00E-04
2	Axon guidance(R)	1.00E-03
2	Signaling by VEGF(R)	1.50E-03
2	VEGF and VEGFR signaling network(N)	1.67E-03
3	Complement cascade(R)	3.33E-04
3	Signaling by the B Cell Receptor (BCR)(R)	5.00E-04
3	Fcgamma receptor (FCGR) dependent phagocytosis(R)	1.00E-03

Supplementary Table 3: Response signature genes

Gene symbol	Gene name	Prediction direction (1: Cell cycle down-regulate, 0: Cell cycle up-regulate)
ATOX1	ATX1 antioxidant protein 1 homolog (yeast)	1
WDR79	WD repeat domain 79	1
ZNF468	zinc finger protein ZNF468	1
NOP17	NOP17	1
OXCT1	3-oxoacid CoA transferase 1	0
TNFRSF21	tumor necrosis factor receptor superfamily, member 21	0
CFDP1	craniofacial development protein 1	0
ANKRD15	Ankyrin repeat domain 15	0
HIGD2A	HIG1 domain family, member 2A	0
GLG1	golgi apparatus protein 1	0

Supplementary Table 4: Summary statistics of pooled neoadjuvant breast cohort

		Number (%)
N	N	895
RS signature	N (%) Up-regulate	298 (33.3)
Age	N (%) ≥ 50	423 (47.3)
ER Status	N (%) Positive	536 (59.9)
Node	N (%) Positive	604 (67.5)
Grade	N (%) Grade = 3	486 (54.3)
Response	N (%) Yes	200 (22.4)

Supplementary Table 5: Univariate and multivariate analysis of the response signature

Factor		Odds Ratio (95% CI)	*p*-value
**Univariable**			
RS signature	Up-regulate vs Down-regulate	2.95 (2.14, 4.08)	< 0.001
Age	≥ 50 vs < 50	0.87 (0.63–1.19)	0.37
ER Status	Positive vs Negative	0.20 (0.15–0.29)	< 0.001
Node	Positive vs Negative	0.97 (0.70–1.36)	0.87
Grade	3 versus 1–2	4.36 (3.00–6.34)	< 0.001
**Multivariable**			
RS signature	Up-regulate vs Down-regulate	1.51 (1.04–2.21)	0.032
Age	≥ 50 vs < 50	0.90 (0.64–1.27)	0.55
ER Status	Positive vs Negative	0.35 (0.23–0.53)	< 0.001
Node	Positive vs Negative	0.74 (0.51–1.07)	0.11
Grade	3 versus 1–2	2.61 (1.72–3.96)	< 0.001

Supplementary Table 6: GSEA of doxorubicin treated MCF7 cells

Curated Gene Set	NES	FDR q-val
SMIRNOV_RESPONSE_TO_IR_6HR_UP	3.36221	< 0.0001
WARTERS_RESPONSE_TO_IR_SKIN	3.11525	< 0.0001
WARTERS_IR_RESPONSE_5GY	3.05664	< 0.0001
**KANNAN_TP53_TARGETS_UP**	2.88044	< 0.0001
**PID_P53DOWNSTREAMPATHWAY**	2.77201	< 0.0001
KERLEY_RESPONSE_TO_CISPLATIN_UP	2.54718	< 0.0001
ZHANG_TLX_TARGETS_DN	2.52436	< 0.0001
SMIRNOV_RESPONSE_TO_IR_2HR_UP	2.50864	< 0.0001
SCHRAETS_MLL_TARGETS_UP	2.43323	< 0.0001
PID_TAP63PATHWAY	2.42225	< 0.0001
GHANDHI_DIRECT_IRRADIATION_UP	2.42023	< 0.0001
KEGG_LYSOSOME	2.41908	< 0.0001
**INGA_TP53_TARGETS**	2.40849	< 0.0001
**ONGUSAHA_TP53_TARGETS**	2.39097	< 0.0001
**KEGG_P53_SIGNALING_PATHWAY**	2.36571	< 0.0001
**AMUNDSON_DNA_DAMAGE_RESPONSE_TP53**	2.32045	< 0.0001
MISSIAGLIA_REGULATED_BY_METHYLATION_UP	2.31993	< 0.0001
**BIOCARTA_P53_PATHWAY**	2.26956	< 0.0001
AMIT_EGF_RESPONSE_120_MCF10A	2.18145	< 0.0001
DACOSTA_UV_RESPONSE_VIA_ERCC3_UP	2.17738	< 0.0001

Supplementary Table 7: p53 probe set signature

Probe set	Description	MUTp53 (0:Correlated. 1: Anti-correlated)
203755_at	budding uninhibited by benzimidazoles 1 homolog beta (yeast), BUB1B	0
201000_at	alanyl-tRNA synthetase, AARS	0
201202_at	proliferating cell nuclear antigen, PCNA	0
218039_at	nucleolar and spindle associated protein 1, NUSAP1	0
207828_s_at	centromere protein F, 350/400 kDa (mitosin), CENPF	0
200754_x_at	serine/arginine-rich splicing factor 2, SRSF2	0
201663_s_at	structural maintenance of chromosomes 4, SMC4	0
202870_s_at	cell division cycle 20 homolog (S. cerevisiae), CDC20	0
200052_s_at	interleukin enhancer binding factor 2, 45 kDa, ILF2	0
208755_x_at	H3 histone, family 3A /// H3 histone, family 3A pseudogene, H3F3A /// LOC440926	0
213453_x_at	glyceraldehyde-3-phosphate dehydrogenase, GAPDH	0
201342_at	small nuclear ribonucleoprotein polypeptide C, SNRPC	0
201464_x_at	jun proto-oncogene, JUN	1
214334_x_at	DAZ associated protein 2, DAZAP2	1
208174_x_at	zinc finger (CCCH type), RNA-binding motif and serine/arginine rich 2, ZRSR2	1
201967_at	RNA binding motif protein 6, RBM6	1
203509_at	sortilin-related receptor, L(DLR class) A repeats-containing, SORL1	1
200804_at	transmembrane BAX inhibitor motif containing 6, TMBIM6	1

Supplementary Table 8: Response characteristics of the validation cohorts

Characteristic	Test cohorts						
	GSE25055	GSE25065	GSE20194	GSE20271	GSE22093	GSE30161	GSE52735
Samples	310	198	278	171	103	58	37
Responders	86	51	56	26	28	19	24
Non-responders	220	131	222	152	69	36	13
N/A	4	16	0	0	6	3	0
Regimen	AT	AT	AT	A or AT	A	Platinum, T	Unknown

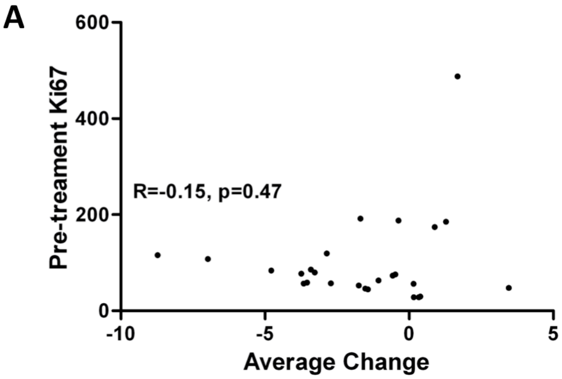

Supplementary Figure 1: (A) Relationship between changes in Module 1 during treatment and pre-treatment levels of ki67 transcript.

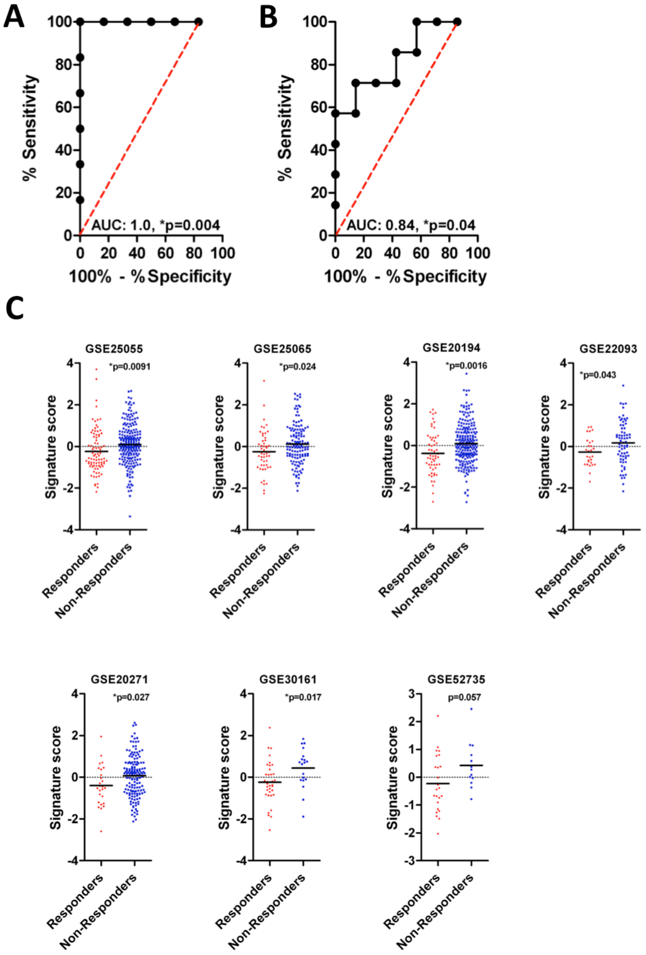

Supplementary Figure 2: ROC analysis of Response Signature (RS). The RS is associated with up-regulation of cell cycle genes in response to chemotherapy in the **(A)** training cohort and the **(B)** validation cohort. **(C)** The RS is associated with chemotherapy response in 5 breast cancer data sets (GSE25055, GSE25065, GSE20194, GSE22093, GSE20271) and an ovarian cancer data set (GSE30161) and a colon cancer data set (GSE52735).

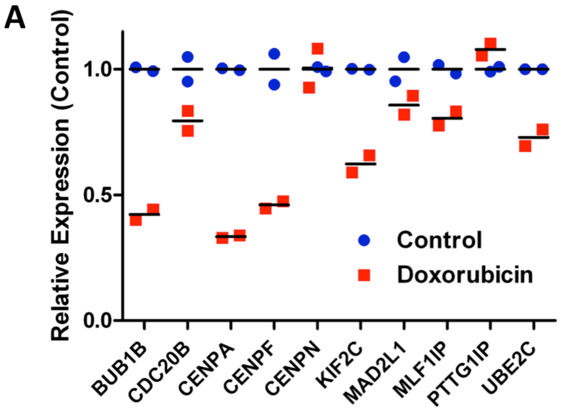

Supplementary Figure 3: (A) Response signature genes are down-regulated in doxorubicin treated MCF7 cells.

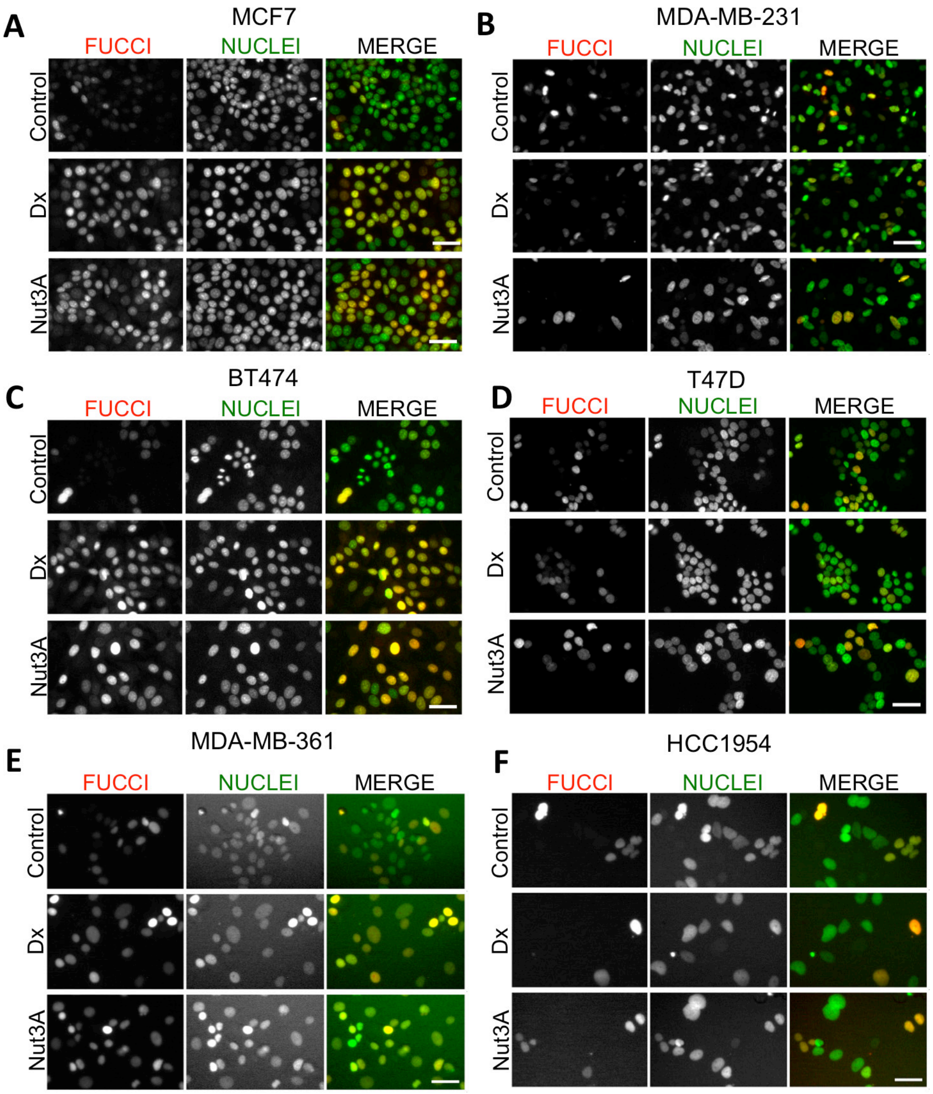

Supplementary Figure 4: (A–F) Micrographs of six human breast cancer cell line in control, doxorubicin or Nutlin3A treatments (24 hours post treatment). Scale bar = 50 um.

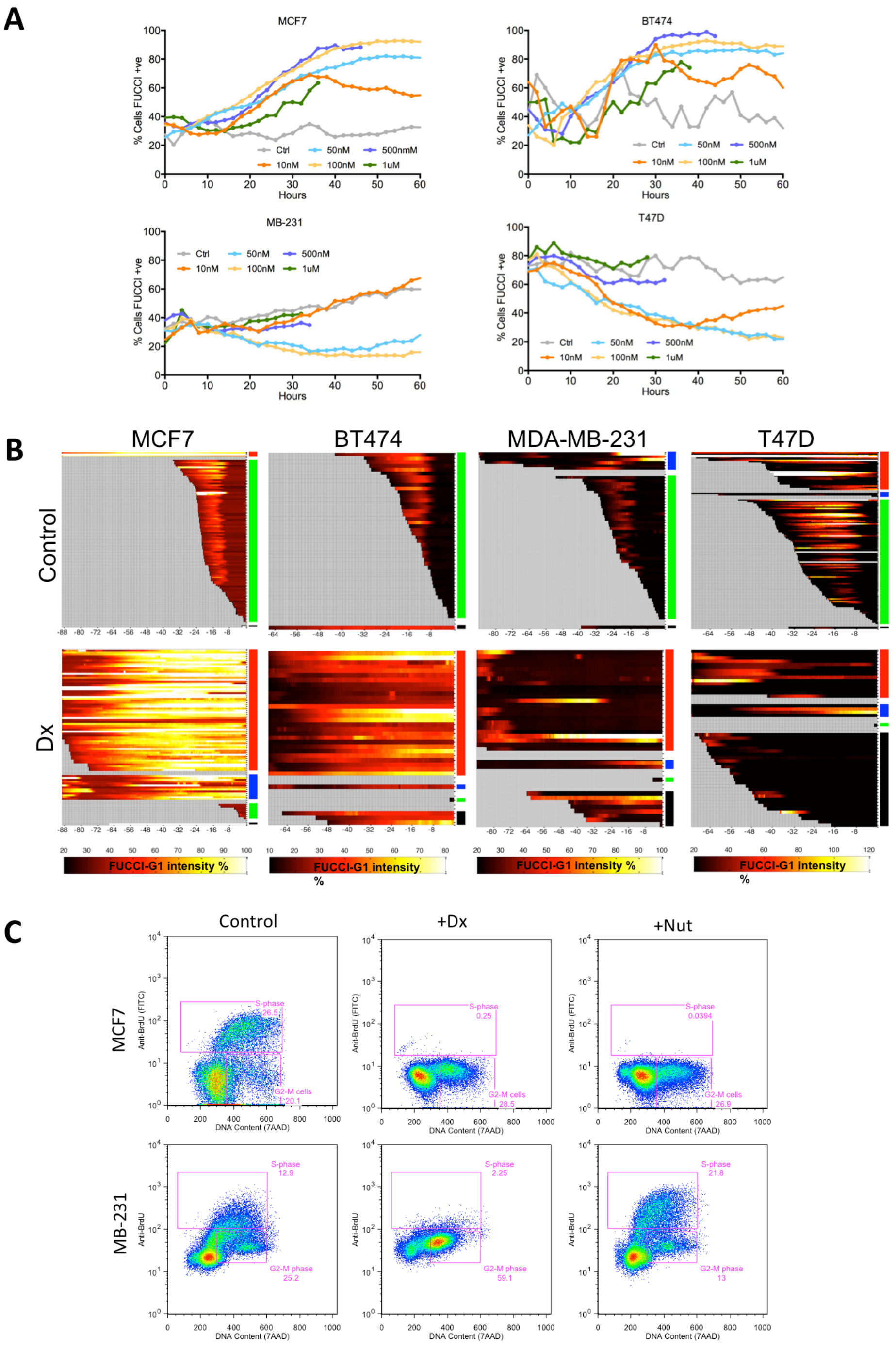

Supplementary Figure 5: (A) Analysis of FUCCI expression response in breast cancer cell lines to a range of Dx doses. **(B)** Single cell tracking of founding cells and their daughters in control and doxorubicin conditions. Each bar represents one cell. X-axis denotes time in hours counting down from the start of tracking until an event occurs (denoted on right Y-axis at 0 hrs; events: green = cell division, red = no division, blue = endoreplication, black = apoptosis). A scale of relative FUCCI-G1 expression intensity is included. **(C)** BrDU incorporation analysis of Dx and Nutlin3A (Nut) treated MCF7 and MDA-MB-231 cells.

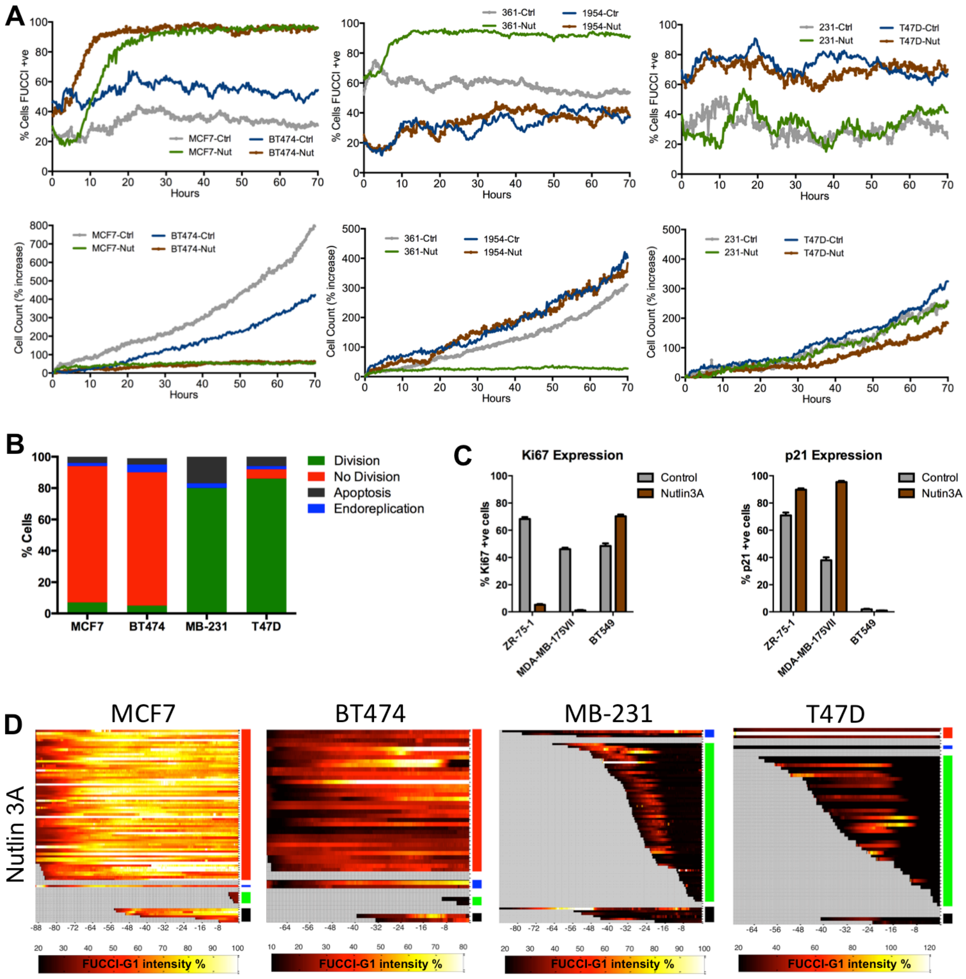

Supplementary Figure 6: The cell cycle response of human breast cancer cell lines to Nutlin3A treatment. (A) Percentage of cells expressing FUCCI-G1 and cell count increase over 3 days in the presence of Nutlin3A (Nut) or control (Ctrl) conditions. **(B)** Summary of single cell tracking of human BC lines in Nutlin3A conditions. Chart depicts the percentage of cells for each line in different stages of the cell cycle. **(C)** Ki67 and p21 staining of Nutlin3A treated cell lines. **(D)** Single cell tracking of founding cells and their daughters in Nutlin3A conditions. Each bar represents one cell. X-axis denotes time in hours counting down from the start of tracking until an event occurs (denoted on right Y-axis at 0 hrs; events: green = cell division, red = no division, blue = endoreplication, black = apoptosis). A scale of relative FUCCI-G1 expression intensity is included.

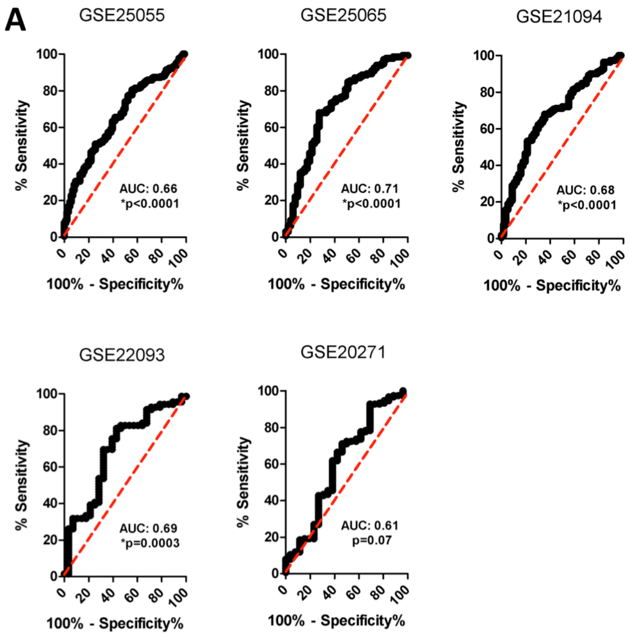

Supplementary Figure 7: **(A)** ROC analysis of the p53 signature in chemotherapy response in 5 breast cancer datasets.
